# Organisational Climate and Pro-environmental Behaviours at Work: The Mediating Role of Personal Norms

**DOI:** 10.3389/fpsyg.2021.635739

**Published:** 2021-09-21

**Authors:** Carla Mouro, Ana Patrícia Duarte

**Affiliations:** ^1^Centro de Investigação e Intervenção Social, ISCTE-Instituto Universitário de Lisboa, Lisboa, Portugal; ^2^Business Research Unit, ISCTE-Instituto Universitário de Lisboa, Lisboa, Portugal

**Keywords:** pro-environmental behaviours, organisational climate, personal norms, descriptive norms, workplace

## Abstract

Organisations are currently strongly encouraged to adopt more responsible production patterns aligned with sustainable development goals (SDGs). Pro-environmental behaviours (PEBs) in the workplace can strengthen the expected positive impacts of organisations’ environmental performance and engender more sustainable transitions to low-carbon production. Research on PEBs at work is relatively recent, so this field still lacks studies of the role of organisational policies and practices in workers’ adoption of these behaviours and of psychosocial processes that contribute to more sustainable workplaces. The present research examined how perceptions of organisations’ environmental policies and practices (i.e., organisational climate or injunctive norms) and of coworkers’ PEBs (i.e., descriptive norms) affect employees’ self-reported voluntary PEBs. Thogersen’s norm taxonomy model was also applied to address the role of personal norms. Self-commitment to sustainable goals at work can play a fundamental role in workers’ behavioural choices, so this research further investigated whether personal norms mediate the relationship between perceived pro-environmental organisational climate and reported workplace PEBs. To test the proposed model, data were collected on 210 workers from different business sectors, who completed an online questionnaire. The analyses showed that, after controlling for the effects of tenure, education level, and a management position, a pro-environmental organisational climate predicts stronger personal norms and a greater tendency to adopt PEBs at work (adjusted *R* squared=0.36), providing evidence of complete mediation. Coworkers’ perceived descriptive norms also contribute directly to self-reported PEBs. The discussion of the results focuses on the importance of organisational level initiatives as a way to promote change in individuals’ behaviours, which can have positive consequences for workplaces’ transition to sustainability.

## Introduction

Organisations are currently strongly encouraged to adopt more responsible production patterns in alignment with the United Nations’ Sustainable Development Goals (SDGs). The SDGs to be achieved by 2030 form a framework calling for urgent action at various levels of sustainability (United Nations, 2015), including paying attention to organisational performance’s impacts on the environment (i.e., SDG12 – responsible consumption and production). Organisations worldwide are increasingly adopting environmental management systems such as the International Organisation for Standardisation’s ISO14000 and the European Union’s Eco-Management and Audit Scheme. These systems are a set of processes and practices that enable organisations to reduce their environmental impacts while increasing operational efficiency. In addition, organisations are implementing other initiatives to reduce their ecological footprint and contribute to safeguarding the environment as part of their social responsibility and sustainability policies [Klynveld Peat Marwick Goerdeler (KPMG), 2017; Duarte et al., 2019; Tian and Robertson, 2019]. Climate change has also become an important challenge in organisations’ operations (Dahlmann et al., 2019), and successfully dealing with this challenge requires all members – whether they are decision makers or workers – to contribute to implementing the relevant policies. These individuals are ultimately responsible for applying environmental practices in daily routines (Daily et al., 2009; Yuriev et al., 2018).

Pro-environmental behaviours (PEBs) in the workplace can contribute to these practices’ positive impacts on organisations’ environmental performance (Tsai et al., 2016) and to a sustainable transition to low-carbon production. PEBs in this context may include behaviours such as conserving energy and resources, reducing waste, increasing recycling, or advocating eco-friendly behaviours to coworkers (Cantor et al., 2015; Dumitru et al., 2016; Saeed et al., 2019; Canova and Manganelli, 2020). Some of these behaviours are similar to PEBs at home, but workplace PEBs does not necessarily have the same predictors (Dumitru et al., 2016; Whitmarsh et al., 2018).

More research is needed on how to incentivise workers to engage in these behaviours at work especially since this context is one in which people spend much of their time. More specifically, this field still lacks studies of the role of organisations’ policies and practices in workers’ adoption of PEBs (Lo et al., 2012; Yuriev et al., 2018) and of the psychosocial processes that contribute to more sustainable workplaces (Ciocirlan et al., 2020). This line of research is important to developing a better understanding of how to encourage PEBs at work (Paillé and Boiral, 2013; Carmeli et al., 2017; Wesselink et al., 2017) and remove potential barriers to their acceptance (Carrico and Riemer, 2011; Yuriev et al., 2018).

### Normative Theories and Workplace PEBs

One of the strongest predictors of behaviours is social norms (McDonald and Crandall, 2015). Social norms are shared expectations about what is appropriate behaviour in specific contexts (McDonald and Crandall, 2015). Within organisations, these norms can be understood as workers’ perceptions of organisational climate (Norton et al., 2014). This climate is traditionally defined as how employees perceive their organisation’s formal policies and practices with reference not only to processes and procedures about which workers are aware but also to patterns they usually observe among coworkers (Schneider, 1990; Schneider et al., 2013). Researchers have found that organisational climate is an important driver of employees’ attitudes and behaviours and have shown that a pro-environmental organisational climate can contribute to workers’ organisational commitment (Tilleman, 2012), work engagement (Hicklenton et al., 2019), organisational identification (Afsar et al., 2018), and job satisfaction (Spanjol et al., 2015). These impacts tend to be stronger for employees who perceive themselves as sharing values and interests with their organisation (Norton et al., 2012; Hicklenton et al., 2019).

A classic distinction made between types of social norms differentiates injunctive norms – what is approved – and descriptive norms – what is observed (Cialdini et al., 1990). Based on this distinction, organisational climate’s components can be divided into two categories: injunctive norms that indicate to workers which environmental concerns are important to their organisation and expected of employees and descriptive norms that correspond to how coworkers behave in the workplace. The former norms thus hold up the organisation as a referent for workers. This situation is hereafter referred to as “a pro-environmental organisational climate.” Descriptive norms are, in contrast, perceptions of coworkers’ PEBs. This assessment is hereafter termed “a pro-environmental coworker climate.” Both types of climate are expected to be connected to employees’ adoption of PEBs, although these climates’ effects can follow different paths to these behaviours.

Norton et al. (2014) adopted the above distinction and sought to determine which climate is more closely associated with different PEBs. The cited authors’ findings suggest that a pro-environmental organisational climate predicts workers’ involvement in task-related PEBs, i.e., behaviours associated with assigned tasks. A pro-environmental coworker climate is, in turn, a better predictor of voluntary PEBs, that is, behaviours that exceed what is officially expected from employees as part of their work (Bissing-Olson et al., 2013). However, these findings imply that formalised systems of environmental procedures and standards have repercussions for task-related PEBs only – a conclusion not fully supported by other studies that have shown that sustainability policies generally promote workplace PEBs (e.g., Paillé and Boiral, 2013; Zientara and Zamojska, 2018; Magill et al., 2020).

The present research sought to address some limitations in a study of Norton et al. (2014). One limitation had to do with the assessment of descriptive norms. The items used focused on what employees value rather than on what behaviours they engaged in as part of their organisation. The current study attempts to clarify the role of descriptive norms by applying an approach similar to that used in social norms research (e.g., Goldstein et al., 2008; Gökeritz et al., 2010; Mouro and Castro, 2016).

Another limitation is related to PEBs’ measurement. Although items of Norton et al. (2014) capture the distinction between task-related and voluntary PEBs, the items’ wording is quite abstract, causing difficulties in terms of understanding the types of behaviour respondents have in mind when they formulate their answers. The present research measured more concrete behaviours, which were selected because they are sufficiently common to occur in different types of organisations, business sectors, and work tasks. The rewritten items also focused more specifically on one type of PEB – voluntary behaviours. This study thus sought to determine whether pro-environmental organisational and coworkers climates predict voluntary workplace PEBs such as saving energy and water, separating waste for recycling or actively promoting these behaviours among colleagues.

### Personal Norms

Previous research has showed that both injunctive and descriptive norms affect behaviour (e.g., Goldstein et al., 2008), but they appear to do so *via* different processes (Thøgersen, 2006). According to Thøgersen (2006) norm taxonomy model, injunctive norms have an effect on behaviours indirectly through personal norms. The latter norms are feelings of obligation and a commitment to engage in specific behaviours (Schwartz, 1977). In general, personal norms are positively related to various PEBs related to resource conservation at home (e.g., Thøgersen, 2006; Castro et al., 2009) and in other areas that are legally regulated (cf. Mouro and Castro, 2016). Scherbaum et al. (2008) and Chou (2014) showed that personal norms are also significant predictors of PEBs in work contexts.

Personal norms are considered a strong predictor of PEBs (Niemiec et al., 2020), yet their influence can be weakened when strong barriers are put up against these behaviours (Thøgersen, 1996). Recent studies have confirmed that employees may not always feel motivated to commit to acting in pro-environmental ways at work, particularly if these individuals believe that these practices should not be considered their responsibility or if workers feel the necessary conditions to complete these tasks do not exist (Greaves et al., 2013; Ruepert et al., 2015). Some studies have also highlighted how personal norms’ role can differ depending on the type of activity involved (Lokhorst et al., 2011). Task-related PEBs may be more closely associated with external instrumental pressures (e.g., salaries and subsidies), while voluntary PEBs can depend more strongly on the internalisation of values that direct individual workers to act in specific ways (Dumitru et al., 2016).

Factors that function as antecedents of personal norms related to being a more environmentally conscious employee thus play an important role in the adoption of PEBs. Previous research has focused on how general environmental values predict personal norms in the workplace (Ruepert et al., 2016). A less frequently explored topic is a pro-environmental organisational climate’s impact on personal norms, namely, employees’ commitment to lessening their work and organisation’s environmental impacts. According to the literature reviewed for the present research, only the study of Zhang et al. (2013) examined the relationship between organisational climate and both personal norms and PEBs at work. However, the cited study focused exclusively on an electricity-saving workplace climate and behaviours, without testing for a mediating effect.

The present research’s first hypothesis thus focused on the mediating role of personal norms in the relationship between pro-environmental organisational climate and PEBs. A basic assumption of Thøgersen (2006) is that, if workers perceive their organisation’s values and actions as environmentally friendly, this perception generates meaningful reflection about these practices (Afsar and Umrani, 2020). In addition, these individuals are more likely to internalise this commitment to preserving the environment as an important dimension of being a good employee. This sense of obligation to become more pro-environmental can then translate into more workplace PEBs (Scherbaum et al., 2008; Chou, 2014). Therefore, the present study’s first hypothesis focuses on a mediating relationship between the above variables, with further details provided by the two subhypotheses:

*H1*: Personal norms regarding being a pro-environmental worker mediate the relationship between environmental organisational climate and voluntary PEBs.
*H1a*: Environmental organisational climate is positively associated to personal norms related to being a pro-environmental worker.
*H1b*: Personal norms related to being a pro-environmental worker are positively linked with voluntary PEBs.

In contrast, descriptive norms, that is, perceptions of what coworkers do in the workplace, have a direct effect on employees’ behaviour (Thøgersen, 2006; Niemiec et al., 2020) and, more specifically, on voluntary PEBs (Norton et al., 2014). The current research’s second hypothesis posited that:

*H2*: Pro-environmental coworker climate is positively associated with voluntary PEBs at work.

## Materials and Methods

### Participants and Procedures

The participants comprised 210 employees that voluntarily filled in an online survey. Their ages ranged from 20 to 66years old (mean=36.6; *SD*=10.8). The majority were females (58.6%) with a higher education degree (72.9%). The respondents worked for organisations operating in Portugal, and 63.8% had a permanent employment contract and 19.5% had a management position. Overall, these workers had a mean tenure of 9.2years (minimum=0.5; maximum=40) in their current organisation. The respondents worked mostly in the tertiary sector (89.2%) in various areas including, among others, consultancy services (13.4%), education (10.0%), commercial services (9.6%), health and social services (9.1%), and information and communication technologies (8.1%). Almost three-quarters of the participants worked for for-profit (71.3%) and private organisations (74.6%). About one-third had jobs in extremely large organisations (32.1%), while a fifth of the sample worked for medium-sized organisations (23.0%).

The survey was conducted using the Qualtrics Surveys online platform, and the participants were recruited *via* social media (i.e., a non-probabilistic convenience sampling technique). The study assumed a cross-sectional correlational design, so the data were collected on the relevant variables at the same time and from the same source. The project followed the ethical standards guidelines of Portugal’s Order of Psychologists, and the respondents were informed about how their responses’ confidentiality and anonymity would be safeguarded.

### Measures

The survey started with the informed consent and then included the four measures presented below. It ended with questions regarding socio-demographic and professional characteristics of respondents.

#### Pro-environmental Organisational Climate

Four items based on research of Turker (2009), Duarte (2011), and Norton et al. (2014) were developed to measure perceptions of organisational climate, namely, organisational policies and practices related to environmental sustainability. The participants rated how much they thought their organisation “makes an effort to reduce its impact on the environment,” “makes an effort to reduce the natural resources used during its functions (e.g., water and energy),” “separates materials and waste for recycling.” and “upholds the importance of protecting the environment.” The responses were given on a scale ranging from 1 (‘totally disagree’) to 5 (‘totally agree’). The four-item scale showed high internal consistency [Cronbach’s alpha (*α*)=0.80], and a mean score was calculated for use in subsequent analyses.

#### Pro-environmental Coworker Climate

Perceived pro-environmental coworker climate was assessed with four items based on studies of Gökeritz et al. (2010) and Carrico and Riemer (2011). The respondents rated how many employees in their organisation “turn off the lights when they leave a room,” “use as little water as possible,” “shut down equipment after using it,” and “separate materials and waste for recycling.” The responses were given on a scale ranging from 1 (“no one”) to 5 (“all workers”). The four-item scale had good internal consistency (*α*=0.76), so a mean score was estimated for use in further analyses.

#### Personal Norms Related to Being a Pro-environmental Worker

Personal norms were measured with five items based on research of Chou (2014) and Mouro and Castro (2016) and adapted to address specifically employees’ commitment to environmental sustainability at work. The participants rated their agreement with the following items. “I feel personally responsible for this organisation’s contribution to environmental issues.” “I worry about being an “environmentally friendly” worker.” “I feel it’s important that the organisation where I work is concerned about the environment.” “I worry about my organisation’s negative impacts on the environment.” “I believe organisations need to commit seriously to protecting nature.” The responses were given on a scale ranging from 1 (“totally disagree”) to 5 (“totally agree”). The five-item measurement instrument showed good internal consistency (*α*=0.77), and a mean score was computed for use in subsequent analyses.

#### PEBs at Work

Pro-environmental behaviours were measured using seven items based on studies of Robertson and Barling (2012), Greaves et al. (2013), and Mouro and Castro (2016). Besides reporting the frequency of the four behaviours measured for pro-environmental coworker climate, the respondents also indicated how often they themselves “defend the importance of engaging in environmentally friendly behaviours,” “offer to participate in environmental protection initiatives promoted by my organisation,” “make suggestions about how my organisation can become more ‘environmentally friendly’.” The responses were given on a scale ranging from 1 (“never”) to 5 (“very frequently”). The seven-item scale had good internal consistency (*α*=0.72), so a mean score was calculated for use in further analyses.

### Common Method Bias

To prevent common method bias, different rating scales were used (Podsakoff et al., 2003). In addition, unrotated principal component analysis was conducted with all the items of the scales used in the present study to check if the adopted measures passed the Harman’s single factor test. This test is a diagnostic technique used to evaluate whether common method variance is a problem (Podsakoff et al., 2003). The analysis showed that the first factor explains less than 50% of the variance, more specifically, 28% attributed to the first factor, with a total of 68% of variance explained (Kaiser-Meyer-Olkin=0.81; *p*<0.001). The results thus confirm that common method bias did not significantly weaken the study’s validity or distort interpretations of the findings.

## Results

Statistical analyses were conducted using IBM SPSS v26 software, and the mediation test was carried out with the macro PROCESS v3.2 programme (Hayes, 2018). Table 1 provides the descriptive statistics and intercorrelations between the model’s variables and relevant socio-professional characteristics. On average, the participants reported that their organisation is moderately involved in environmentally significant policies and practices (mean=3.62; *SD*=0.81) and that some coworkers voluntarily adopt PEBs at work (mean=3.35; *SD*=0.73). The respondents also described themselves as having strong personal norms regarding being pro-environmental workers (mean=4.03; *SD*=0.57) and a moderately high level of voluntary adoption of workplace PEBs (mean=3.70; *SD*=0.65).

**Table 1:** Descriptive statistics, correlations, and internal consistency for variables (number=210).

		*M*	*SD*	1	2	3	4	5	6	7	8
1.	Tenure	9.15	9.34								
2.	Education	–	–	−0.24^**^							
3.	Management position	–	–	0.28^**^	0.03						
4.	Organisation size	–	–	0.07	0.01	−0.10					
5.	Coworker climate	3.35	0.73	0.12	−0.03	0.07	−0.19^**^	(0.76)			
6.	Organisational climate	3.62	0.81	0.18^**^	−0.13	0.17^*^	0.02	0.42^**^	(0.80)		
7.	Personal norms	4.03	0.57	0.19^**^	0.09	0.18^*^	−0.02	0.27^**^	0.34^**^	(0.77)	
8.	PEBs@work	3.70	0.65	0.22^**^	0.15^*^	0.21^*^	−0.19^**^	0.42^**^	0.32^**^	0.49^**^	(0.72)

*M*, mean.

* *p*<0.05;

** *p*<0.01.

Management position was scored 0 for “no” and 1 for “yes”; organisation size was scored as 1 for micro (up to nine workers), 2 for small (10–49 workers), 3 for medium-sized (50–249 workers), 4 for large (250–500 workers), and 5 for extremely large (more than 500 workers); Spearman’s rho was used to calculate correlations; Cronbach’s *α* in parenthesis.

Spearman’s correlation coefficients were computed because dichotomous variables were present. Both pro-environmental organisational and coworker climate, as well as personal norms, are positively associated with PEBs. Participants’ gender, age, and type of employment contract (0=permanent; 1=non-permanent) are not significantly related to the criterion variable. Tenure, level of education, a management or non-management position, and organisation size were significantly related to adopting PEBs in the workplace, so these factors were included as covariates in subsequent analyses. To test the direct and indirect effects proposed in the hypotheses, a mediation analysis was conducted using macro PROCESS’s Model 4 (Hayes, 2018). Tolerance (≥0.74) and variance inflation factor (VIF) values (≤1.10) had been previously checked to ensure multicollinearity did not exist between variables. Both values are within the recommended thresholds, exceeding the cut-off point of 0.10 for tolerance (Cohen et al., 2003) and falling below 5.00 for VIF (Montgomery and Peck, 1982).

The first hypothesis focused on the expected positive relationship between employees’ perceptions of a pro-environmental organisational climate and their reported PEBs *via* personal norms. The results confirm that pro-environmental organisational climate significantly predicts employees’ personal norms in favour of PEBs (*B*=0.16; 95% CI=0.07; 0.26), and personal norms also significantly predicts reported levels of PEBs (*B*=0.31; 95% CI=0.17; 0.46), thus supporting subhypotheses H1a and H1b, respectively (Table 2). In addition, pro-environmental organisational climate’s indirect effect is statistically significant, which provides evidence of a mediation effect (*B*=0.05; 95% CI=0.01; 0.06). Hypothesis H1 was thus confirmed.

To determine if the mediation effect was full or partial, the total and direct effects of a pro-environmental organisational climate on PEBs were compared. This climate’s total effect on PEBs is significant (*B*=0.12; 95% CI=0.00; 0.22), suggesting that organisations’ investment in environmentally sustainable practices directly contributes to workers’ voluntary PEBs. This climate’s direct effect, however, is not statistically significant (*B*=0.07; 95% CI=−0.04; 0.17), confirming that this relationship is fully mediated by personal norms. The results, therefore, indicate that a pro-environmental organisational climate reinforces employees’ personal commitment to their organisation’s sustainability. This commitment subsequently generates more voluntary PEBs at work (Table 2).

**Table 2:** Total, direct, and indirect effects.

	Personal norms			PEBs@work		
	*B*	LLCI	ULCI	*B*	LLCI	LCI
Total effect
Constant				1.56^**^	0.87	2.25
Organisational climate				0.12^*^	0.01	0.22
Coworker climate				0.24^**^	0.12	0.36
Management position				0.14	−0.06	0.33
Tenure				0.02^**^	0.01	0.03
Education				0.27^**^	0.14	0.40
Organisation size				−0.07^**^	−0.13	−0.02
				*F*(6,202)=14.65; *p* <0.000; *R^2^* =0.30		
Direct effect
Constant	2.21^**^	1.57	2.85	0.87^*^	0.14	1.50
Organisational climate	0.16^**^	0.07	0.26	0.07	−0.04	0.17
Personal norms				0.31^**^	0.17	0.46
Coworker climate	0.14^*^	0.03	0.25	0.20^**^	0.08	0.31
Management position	0.08	−0.11	0.26	0.11	−0.08	0.30
Tenure	0.01^**^	0.00	0.02	0.01^**^	0.00	0.02
Education	0.17^**^	0.05	0.29	0.22^**^	0.09	0.35
Organisation size	0.01	−0.05	0.06	−0.08^**^	−0.13	−0.02
	*F*(6,202)=9.08; *p* <0.000; *R^2^* =0.21			*F*(7,201)=16.35; *p* <0.000; *R^2^* =0.36		
						
		Effect		BootLLCI		BootULCI
Indirect effect		0.05		0.01		0.10

*B*, non-standardised coefficients.

* *p*<0.05;

** *p*<0.01.

LLCI, lower limit CI; ULCI, upper limit CIs; management position was scored 0 for “no” and 1 for “yes”; organisation size was scored as 1 for micro (up to nine employees), 2 for small (10–49 employees), 3 for medium-sized (50–249 employees), 4 for large (250–500 employees), and 5 for extremely large (more than 500 employees).

The second hypothesis posited that a perceived pro-environmental coworker climate would be positively related to reported workplace PEBs. This hypothesis was corroborated by the results (*B*=0.20; 95% CI=0.08; 0.31). The model overall explains 36% of the variance in workers’ PEBs at work [*F*(6, 201)=16.33; *p*<0.001].

## Discussion

Pro-environmental behaviours in the workplace have received increased attention in the literature in recent years. Organisations are acknowledging their responsibility and central role in the transition to sustainability, and they have launched multiple initiatives [Klynveld Peat Marwick Goerdeler (KPMG), 2017] – some aimed at reducing their business activities’ environmental impacts. As is true of many other organisational policies, these initiative’s success depends largely upon organisational members’ collaboration (Daily et al., 2009). The latter are responsible for executing daily the processes, procedures, and actions defined by top-level decision makers. More research has thus been conducted to understand more fully how organisations can motivate their employees to behave in pro-environmental ways. Scholars have also increasingly sought to analyse the psychosocial processes that sustain PEBs at work, often based on models and studies of PEBs at home (Whitmarsh et al., 2018).

The present research sought to contribute to this endeavour by investigating whether a pro-environmental organisational climate predicts workers’ involvement in workplace voluntary PEBs *via* a strengthening effect on personal norms. The first hypothesis was supported by a significant mediation effect, indicating that, when organisations invest in pro-environmental policies and practices, these reinforce workers’ personal commitment to becoming pro-environmental. In turn, this personal norm increases the adoption of PEBs at work. These findings corroborate previous studies showing that personal norms – whether general (Zhang et al., 2013; Chou, 2014) or focused on work contexts (Ruepert et al., 2016; Afsar and Umrani, 2020; Ciocirlan et al., 2020) – contribute to workplace PEBs. The current results extend the existing literature by showing that a pro-environmental organisational climate can function as an injunctive norm that incentivises employees’ personal commitment to behaving in sustainable ways, which is in line with a theoretical model of Thøgersen (2006).

More concretely, the present findings extend previous research on the association between a perceived pro-environmental organisational climate and PEBs at work (Norton et al., 2014) by confirming that this injunctive normative influence can also be associated with voluntary PEBs – and not just task-related PEBs – *via* personal norms. As previously highlighted in the literature, personal norms can be particularly important to activating voluntary PEBs at work (Lokhorst et al., 2011; Dumitru et al., 2016). Acting pro-environmentally would be, in this case, rewarded by “doing the right thing” – the internalised sense of obligation that characterises these norms – more than by the external rewards associated with task-related PEBs. The current results, therefore, contribute to clarifying one of the psychosocial processes through which organisational sustainability initiatives can affect employees’ pro-environmental organisational citizenship behaviours (Paillé and Boiral, 2013).

The second hypothesis stated that pro-environmental coworker climate (i.e., what workers believe others do) has a positive relationship with voluntary PEBs at work. This hypothesis was also corroborated, a result which is in line with a research of Norton et al. (2014). The present findings also corroborate theoretical frameworks of Cialdini et al. (1990) and Thøgersen (2006), which state that descriptive norms have a direct effect on behaviours (Gökeritz et al., 2010).

### Limitations and Future Research

This study had some limitations that need to be considered when interpreting its results. First, the research relied on self-reported data provided by employees recruited to form a non-probabilistic sample through a convenience sampling approach. Additional studies are required to confirm the findings’ robustness by using other sampling methods, surveying workers from specific organisations, or selecting context-specific behaviours. Further research should also focus on gaining a better understanding of the impacts of injunctive and personal norms in different sectors of activity. Previous studies have suggested that personal norms, in particular, can be less closely associated with pro-environmental practices in the primary sector (Niemiec et al., 2020). This pattern could be due to, for instance, farms being a more individualised work setting (Lokhorst et al., 2011; Caffaro et al., 2019) or institutional pressures to engage in PEBs (e.g., environmental laws and subsidies) being resisted or taking more time to be internalised as personal values (Mouro and Castro, 2016, 2017).

Second, the present study’s type of measurement needs to be complemented with other methods for assessing both workplace climate (e.g., using more than one data source and a criteria matrix to analyse and classify organisational policies and practices) and behaviours (e.g., observational data on waste separation). In addition, the present results show that employees’ beliefs about coworkers’ normative conduct may be affected by the size of the group being evaluated. More specifically, the findings indicate that, in smaller organisations, workers tend to perceive others as acting more often in environmentally friendly ways. This pattern could be related to norm specificity’s effect (Mertens and Schultz, 2021). That is, proximal groups in terms of spatial proximity or shared attributes may be more important than distal, more generic groups to individuals assessing a given norm (Goldstein et al., 2008). More research is needed to understand more fully this effect’s magnitude, including considering additional referents for larger organisations (e.g., departments) to help clarify norm specificity’s role.

Third, another limitation was generated by the correlational research design. Although the mediation analysis (Hayes, 2018) included a directional test of the hypotheses and controlled for systematic errors related to multiple regressions, the model remained recursive, so the variables’ causal relationships are still unclear. For instance, organisations that have invested more in environmental policies and in a reduction of production’s impacts may also be more likely to recruit workers who value these organisational attributes since recruiters might rely on green human resource management practices (Guerci et al., 2016; Saeed et al., 2019). In this case, personal norms would be related to a perceived pro-environmental climate *via* the person-organisation fit (Hicklenton et al., 2019).

Last, the interpretation of mediation effects was limited by a cross-sectional design, in which the entire dataset was collected at the same time from the same source. To prevent the occurrence of common source bias, the present study used different rating scales (Podsakoff et al., 2003). The results of Harman’s single factor test combined with the weak to moderately strong intercorrelations between the variables under study provide some assurance that common source bias was avoided. However, further studies are needed to address this limitation by adopting a longitudinal design, collecting data at different points in time and/or using multiple sources.

This research examined the role of a pro-environmental climate at the organisational and coworker level in predicting employees’ involvement in workplace PEBs. Future studies could also consider the effects of pro-environmental managers’ behaviour since previous research has underlined the importance of leading by example (Ramus and Steger, 2000; Robertson and Barling, 2012; Boiral et al., 2015; Wesselink et al., 2017) to PEBs at work. Non-exemplary leaders’ role in discouraging PEBs is a significant barrier to these behaviours at work (Yuriev et al., 2018). Another possible avenue of research is related to the inclusion in the present model of variables from the theory of planned behaviour (Ajzen, 1991), which has frequently been used to develop theoretical frameworks for research on PEBs (Wesselink et al., 2017; Canova and Manganelli, 2020; Yuriev et al., 2020; Carrus et al., 2021), especially perceived behavioural control. This factor refers to the extent to which workers feel they are sufficiently in control to be able to perform specific kinds of behaviour in particular contexts. Employees’ perceived lack of control can interfere in their ability to adopt PEBs at work (Greaves et al., 2013). Exploring how positive and negative emotions have a role in adopting PEBs at the workplace is another relevant avenue of research, based on recent reviews showing emotions are important predictors of energy saving behaviours (Carrus et al., 2021).

### Practical Contributions

The present study’s findings make practical contributions related to how workplace perceptions can have a normative effect that facilitates the adoption of voluntary PEBs on the job. The results highlight how organisations need to not only promote pro-environmental initiatives and policies but also give more visibility to workplace PEBs in which employees voluntarily engage. Large organisations might experience difficulties in translating their commitment to environmental concerns into everyday practices and supporting contexts (Dumitru et al., 2016). Leaders’ role can be crucial in implementing good communication strategies for disseminating injunctive norms (Robertson and Barling, 2012) and motivating workers and teams to share their commitment to environmentally friendly performance (i.e., descriptive norms).

As each type of norm or climate dimension has a differential impact on behaviour, organisations can follow both paths to encourage voluntary PEBs more fully at work. For example, conservation behaviours (e.g., reducing energy consumption and increasing recycling) are considered to be low-intensity behaviours, with low costs for workers and organisations, but these behaviours are also characterised by low visibility (Ciocirlan et al., 2020). Measures that increase the visibility of coworkers’ descriptive norms can include developing shared goals and communicating achievements through feedback (e.g., Carrico and Riemer, 2011; Dixon et al., 2014). To activate or strengthen personal norms, expectations about workers’ contribution to their organisation’s environmental performance can be made more explicit, for instance, through green human resource management practices (Guerci et al., 2016; Saeed et al., 2019).

In conclusion, the present findings help clarify the importance of organisations’ investment in environmental policies and initiatives as these appear to contribute to workers’ personal commitment to behaving pro-environmentally at work. Employees respond to their environmentally responsible organisation’s efforts by engaging in more voluntary PEBs in their workplace. More sustainable production and a faster transition to sustainability rely on organisations’ ability to rally their workers around these causes, leading by example, defining goals and making already good green practices more visible.

## Data Availability Statement

The raw data supporting the conclusions of this article will be made available by the authors, without undue reservation.

## Ethics Statement

Ethical review and approval was not required for the study on human participants in accordance with the local legislation and institutional requirements. The patients/participants provided their written informed consent to participate in this study.

## Author Contributions

CM and AD formulated the study, designed the data collection, performed the analysis, and wrote this article. All authors contributed to the article and approved the submitted version.

## Funding

This research was partially supported by the Fundação para a Ciência e Tecnologia, Portugal, through Grants UID/GES/00315/2013 and UID/PSI/03125/2013, and contracts DL 57/2016/CP1359/CT0006 and DL 57/2016/CP1359/CT0004.

## Conflict of Interest

The authors declare that the research was conducted in the absence of any commercial or financial relationships that could be construed as a potential conflict of interest.

## Publisher’s Note

All claims expressed in this article are solely those of the authors and do not necessarily represent those of their affiliated organizations, or those of the publisher, the editors and the reviewers. Any product that may be evaluated in this article, or claim that may be made by its manufacturer, is not guaranteed or endorsed by the publisher.
